# The role of DNA methylation in progression of neurological disorders and neurodegenerative diseases as well as the prospect of using DNA methylation inhibitors as therapeutic agents for such disorders

**DOI:** 10.1016/j.ibneur.2022.12.002

**Published:** 2022-12-12

**Authors:** Yousef Rasmi, Ameneh Shokati, Amber Hassan, Shiva Gholizadeh-Ghaleh Aziz, Sepideh Bastani, Ladan Jalali, Faeze Moradi, Shahriar Alipour

**Affiliations:** a Cellular and Molecular Research Center, Cellular and Molecular Medicine Institute, Urmia University of Medical Sciences, Urmia, Iran; b Department of Biochemistry, Faculty of Medicine, Urmia University of Medical Sciences, Urmia, Iran; c Department of Tissue Engineering and Applied Cell Sciences, School of Advanced Technologies in Medicine, Tehran University of Medical Sciences, Tehran, Iran; d Multiple Sclerosis Research Center, Neuroscience Institute, Tehran University of Medical Sciences (TUMS), Tehran, Iran; e Research Institute for Oncology, Hematology and Cell Therapy, Shariati Hospital, Tehran University of Medical Sciences, Tehran, Iran; f Dipartimento di Oncologia ed Emato-Oncologia, Università Degli Studi Di Milano, Via Festa del Perdono, Milano, Italy; g Laboratory of Translatonal Neurosciences, European School of Molecular Medicine, CEINGE Biotecnologie Avanzate S.c.a.rl.Via Gaetano Salvatore, Naples, Italy; h Stem cell and Regenerative Medicine Institute, Tabriz University of Medical Sciences, Tabriz, Iran; i Tissue Engineering and Applied Cell Sciences Division, Department of Anatomical Sciences, Faculty of Medical Sciences, Tarbiat Modares University, Tehran, Iran; j Student Research Committee, Urmia University of Medical Sciences, Urmia, Iran

**Keywords:** DNA methylation, Neurological disorders, Alzheimer’s, Depression, ADHD, Rett syndrome

## Abstract

Genome-wide studies related to neurological disorders and neurodegenerative diseases have pointed to the role of epigenetic changes such as DNA methylation, histone modification, and noncoding RNAs. DNA methylation machinery controls the dynamic regulation of methylation patterns in discrete brain regions.

**Objective:**

This review aims to describe the role of DNA methylation in inhibiting and progressing neurological and neurodegenerative disorders and therapeutic approaches**.**

**Methods:**

A Systematic search of PubMed, Web of Science, and Cochrane Library was conducted for all qualified studies from 2000 to 2022.

**Results:**

For the current need of time, we have focused on the DNA methylation role in neurological and neurodegenerative diseases and the expression of genes involved in neurodegeneration such as Alzheimer's, Depression, and Rett Syndrome. Finally, it appears that the various epigenetic changes do not occur separately and that DNA methylation and histone modification changes occur side by side and affect each other. We focused on the role of modification of DNA methylation in several genes associated with depression (NR3C1, NR3C2, CRHR1, SLC6A4, BDNF, and FKBP5), Rett syndrome (MECP2), Alzheimer's, depression (APP, BACE1, BIN1 or ANK1) and Parkinson's disease (SNCA), as well as the co-occurring modifications to histones and expression of non-coding RNAs. Understanding these epigenetic changes and their interactions will lead to better treatment strategies.

**Conclusion:**

This review captures the state of understanding of the epigenetics of neurological and neurodegenerative diseases. With new epigenetic mechanisms and targets undoubtedly on the horizon, pharmacological modulation and regulation of epigenetic processes in the brain holds great promise for therapy.

## 1 Introduction

The neurological and neurodegenerative disorders, including many sporadic and hereditary disorders, are characterized by the progressive loss of neurons’ structure and function, often associated with neuronal death. The causes of neurological and neurodegenerative diseases are complicated and related to many factors, such as age, heredity, lifestyle, and environmental factors (Gilmore et al., 2008, Re et al., 2012).

Among epigenetic components, DNA methylation could be a significant epigenetic marker that has been most broadly examined (Lu et al., 2013a). DNA methylation is a post-replication alteration that frequently happens in cytosines of the CpG dinucleotide sequence, leading to the exchange of a methyl group from S-adenyl methionine to a cytosine (Jin and Liu, 2018). When DNA is symmetrically methylated, the methyl groups alter DNA structure. The main consequence of methyl alteration is that a variety of transcription factors cannot recognize the DNA and hence induce repression of transcription (Prokhortchouk and Defossez, 2008). DNA methylation in the mammalian nervous system regulates neural stem cell fate, brain development native function, neurodevelopmental disorders, and neurodegenerative diseases (Hirabayashi and Gotoh, 2010, Urdinguio et al., 2009, Xu and Li, 2012). DNA methylation is also associated with memoryand ischemia-induced damage (Xu and Li, 2012). Recent studies reported that DNA methylation changes are associated with cancer and neurological disorders (Lu et al., 2013a, Xu and Li, 2012). These epigenetic modifications regulate the networks of essential genes that mediate physiological processes and represent a simple and rational method to prevent or even treat these disorders (Landgrave-Gómez et al., 2015). New evidence suggests that altering metabolism through exercise or a variety of diets such as ketogenic diets, low-carbohydrate diets, and intermittent fasting can change the concentrations of various metabolites, some of which may modulate the activity of proteins that causes epigenetic modifications (Shimazu et al., 2013, Shyh-Chang et al., 2013).

In this brief overview, we accompanied the emergence of a new understanding of DNA methylation mechanisms and their implications for CNS function and dysfunction. Research in the previous two decades discovered an emerging outline of the relationship between numerous epigenetic pathways and neurological or neurodegenerative disorders. We will begin by outlining the epigenetics and DNA methylation, then focus intensively on recent progress made in the study of DNA methylation in major neurological disorders such as schizophrenia, depression, attention deficit hyperactivity disorder (ADHD), Alzheimer’s disease (AD), and Rett syndrome, as well as the role of DNA methylation in therapeutic approaches for the treatment of these disorders.

## 2 Epigenetics and DNA methylation

**Fig. 1:**
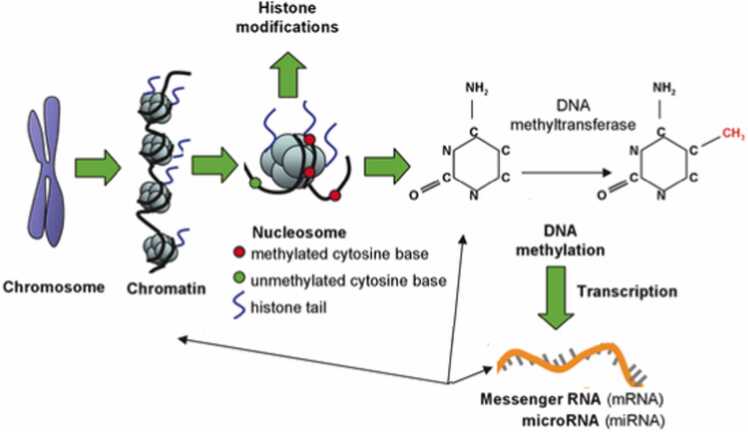
Schematic diagram of Epigenetics Modification.

Epigenetics refers to mechanisms that regulate gene expression without altering the primary DNA sequence. Epigenetic changes affect gene activation in response to environmental cues, which is essential for the primary cell and tissue differentiation (Iridoy Zulet et al., 2017, Abdi et al., 2018). According to epigenetic theory, the genome and the environment can work together to influence regulatory mechanisms that control gene expression by modifying epigenetic DNA marks that can persist for a lifetime (Weinhold, 2006, Kanherkar et al., 2014). Furthermore, the stochastic accumulation of epigenetic changes is linked to aging (Huidobro et al., 2013) as well as sporadic neurological disorders (Wang et al., 2008), for which aging is currently recognized as a significant risk factor (Nussbaum and Ellis, 2003). Human cells undergo epigenetic changes throughout their lives, as previously stated. In identical twins with the same hereditary load, diverse epigenetic patterns are accumulated depending on the environmental factors they are exposed to, for example, diet, tobacco, or exercise. This causes discernible differences in the phenotypes of both twins, indicating different susceptibilities to disease or disease outcomes (Fraga et al., 2005). DNA methylation, histone modification, and noncoding RNA action are all critical epigenetic mechanisms (Fig. 1). DNA methylation is the most studied epigenetic mark, and its relationship to disease development has been extensively researched (Iridoy Zulet et al., 2017). The DNA methylation process is a reversible mechanism wherein methyl groups (–CH3) are delivered to cytosines positioned in CpG (5′-Cytosine-phosphate-guanosine-3′) nucleotides turning these cytosines into 5-methylcytosines (5mC) (Martínez-Iglesias et al., 2020, Alipour et al., 2020). DNA methylation is catalyzed by specific enzymes known as de novo DNA methyltransferases (DNMTs), and it occurs at the expense of ATP and S-adenosylmethionine as methyl donors (Moore et al., 2013).

DNA methylation is an essential part of the epigenetic system, which organizes changes in numerous genes and helps control the expression of genes in all vertebrates (Altuna et al., 2019a). Most cytosine methylation occurs in cytosine phosphate guanine (CpG) islands, which are found in both eukaryotes and prokaryotes. Currently, five states of the cytosine base are known: 5-carboxylcytosine (5cC), 5-formylcytosine (5fC), 5-Hydroxymethylcytosine (5hmC), 5-methylcytosine (5mC), and unmodified cytosine (C). After the unaltered form, the most prevalent state of cytosine in the brains is 5mC, which is mainly found in the CpG dinucleotides. CpG islands are found in more than 60% of mammalian gene promoters (Prasad and Jho, 2019). 5mC was assumed to be related to the suppression of gene expression, while 5hmC, which causes DNA demethylation, was associated with enhanced gene expression. As more study is done, the role of methylation in gene expression depends on the CpG region in the genome. Most of the time, methylation at the gene’s promoter is negatively associated with gene expression (Ogino et al., 2009, Aziz et al., 2020).

DNMTs are tissue- and cell-specifically expressed during neural development as well as in active neurogenesis (Feng et al., 2007) and adult stem cell niches (Kang, 2011), where they have been implicated in neural plasticity and survival (Ooi et al., 2007). Once methylation is established, proteins from the methyl-CpG-binding domain (MBD) family are recruited in methylated loci to stimulate the recruitment of histone modulatory variables (Jones and Takai, 2001, Klose and Bird, 2004), indicating a synergistic modulation of numerous epigenetic marks (Mehler, 2008).

The MBD proteins are also recruited in brain development functions in adults (Chahrour et al., 2008). The most common consequence of DNA methylation is the silencing of genes and noncoding genomic regions, mainly when gene promoters are influenced (Chahrour et al., 2008, Yasui et al., 2007).

This epigenetic process is widespread in brain cells. The results of previous studies indicated that 5hmC is distinctly different from 5mC in its chromatin dependence during neural stem cell (NSC) development. 5-hydroxymethylcytosine (5hmC) has been proposed that it is both an intermediate state in the demethylation process and a significant epigenetic impact on neurological disorders (Chen et al., 2014, Cheng et al., 2015). But in general, the process of DNA demethylation and the enzymes that catalyze this reaction, although DNA demethylases such as cytidine deaminase caused by activation, are only partially understood after the past decade (Bhutani et al., 2010) or the DNA demethylating activity of TET1 (a member of TETs) (Tahiliani et al., 2009) have been identified.

Evidence demonstrates the critical role of DNA methylation in common diseases. Researchers have attempted to use DNA methylation as a biomarker to distinguish epigenetic changes related to disease status, including neurological disorders (Jin and Liu, 2018). In neurons and the nervous system, the overall balance between DNA methylation, demethylation and hydroxymethylation creates different neural patterns in processes such as learning or memory, and their dysregulation may be associated with neurological disorders (Wang et al., 2008).

### 2.1 DNA methylation in neurological disorders

Epigenetic modifications form long-lasting cellular memories in the brain, which are used to translate the mechanisms and responses to environmental stimuli (Akyürek et al., 2021, Kim and Kaang, 2017). Microglia, a type of immune cells, populate the central nervous system (CNS) during early fetal development and can self-renew locally. They remain in the CNS microenvironment throughout life, accounting for 10–15% of all CNS cells (Li and Barres, 2018). There are generally accepted classifications of these cells in the brain in response to infection or tissue injury, pro-inflammatory phenotype (M1-phenotype), and in response to neurodegenerative diseases, the anti-inflammatory phenotype (M2-phenotype) (Nimmerjahn et al., 2005, Xu et al., 2015). Neuron-microglia crosstalk in the CNS affects homeostasis and neuronal function in the healthy brain, so the differentiation of specific phenotypes and activation states of these phenotypes play an essential role in CNS health and disease (Esteller, 2008, Petralla et al., 2021). As a result of neuronal stimulation, several transcription factors cause epigenetic revolutions in microglia, followed by chromatin remodeling and the formation of a distinct microglial phenotype, which may have implications for neuronal activity, maturation, and synaptic networks. Inflammatory status and tissue-specific Transcription Factors such as PU.1, CEBP, IRF8, SMAD2/3, and SALL1 (Holtman et al., 2017) are examples that promote the expression of *IL-6* and tumor necrosis factor (*TNF*), *NF-κB*, *NF-AT,* and *STAT1/3* and cause the histone H3 K4monomethylated (*H3K4me1*) and histone H3 K9lysine acetylation (*H3K9ac*). So this procedure is responsible for gene expression enhancers and chromatin being modified (Holtman et al., 2017, Veremeyko et al., 2019).

Abnormal DNA methylation patterns are associated with a wide range of human neurological diseases, including several neuropsychiatric illnesses (schizophrenia, depression, ADHD), neurodevelopmental disorder (Rett syndrome), neurodegenerative disease (Alzheimer's disease), and cognitive impairment (Qureshi and Mehler, 2013, Weng et al., 2013).

Recent research has revealed a link between DNA methyltransferases and pain processing. Following a peripheral nerve injury, the level of DNA methyltransferases, DNMT3a and DNMT1, increased in the dorsal root ganglia (DRG). By stimulating DRG, these enzymes can promote the gene Kcna2, which influences the voltage-gated potassium channel, resulting in spinal cord sensitization and neuropathic pain symptoms (Sun et al., 2019, Zhao et al., 2017). A decrease in DNA methyltransferases has been detected in various CNS disorders, affecting the BDNF DNA methylation status in the hippocampus, which modulates learning and memory (Guo et al., 2011, Nguyen et al., 2007). Post-mitotic neurons and glial cells make up a large portion of the brain’s cells, both of which have a limited ability to divide. Mature neurons have DNMT1 and DNMT3A expressions. In both development and disease, DNMT3B is required for the dynamic programming of epigenetic regulation (Gao et al., 2020, Ng et al., 1999).

Mutations in *DNMT1* have been identified in hereditary sensory and autonomic neuropathy type 1 (HSAN1) syndrome, with other neuropathies and autosomal dominant cerebellar ataxia, deafness, and narcolepsy (ADCA-DN) (Sun et al., 2014, Kernohan et al., 2016). DNMT1 activity is critical for maintaining DNA methylation, chromatin stability, and gene regulation. Thus, the mutation of DNMT1 impairs DNA methyltransferase activity and decreases heterochromatin binding in the G2 cell cycle stage, resulting in extensive hypomethylation and local hypermethylation (Jin and Robertson, 2013). This may explain its complex pathogenesis in the nervous system. In addition, the decline in the DNMT3a2 expression in the hippocampus has been associated with intellectual and cognitive disorders indicating the essential role of DNMT3a2 in memory formation and mental function (Klein et al., 2011). The results of one study determined that the process of amyotrophic lateral sclerosis- mesenchymal stromal cells (ALS-MSCs) can be modulated by inhibiting overexpressed DNMTs. This approach may provide better efficacy in stem cell therapy (Oh et al., 2016). Another study also showed that methyltransferase levels increased in the demyelinated hippocampus of multiple sclerosis patients, while demethylation enzymes decreased (Chomyk et al., 2017).

DNA methylation can be recognized by a variety of methyl-CpG-binding domain (MBD) proteins “epigenome readers” such as methyl-CpG binding protein 2 (MECP2) and methyl-CpG-binding domain proteins 1–4 (MBD1–4). MECP2 is an X-linked gene that codes for a nuclear protein that binds to methylated DNA and acts as a broad suppressor interacting with histone-modifying complexes (Goffin et al., 2011). Mutations in the MECP2 gene and subsequently irregular expression of MECP2 are the leading cause of the Rett syndrome, a neurological X-linked disorder mainly affecting females. MECP2 mutations have also been associated with a wide range of other neurodevelopmental diseases, including X-linked mental retardation, and autism, representing that mutation in MECP2 has extensive consequences and leads to various neurodevelopmental disorders (Bostick et al., 2007, Amir et al., 1999, Gonzales and LaSalle, 2010).

Abnormal DNA methylation patterns have also been reported in several neurodegenerative disorders. Alzheimer’s disease (AD) is a common neurodegenerative illness marked by progressive dementia that may be due to abnormal DNA methylation. In some AD brain research, elevated levels of S-adenosylhomocysteine have been detected, which inhibits DNMT s activity (Kennedy et al., 2004). Furthermore, DNA hypomethylation has been identified, in promoter CpGs of AD-related genes such as presenilin 1(PS1), APP, and β-site APP-cleaving enzyme1 (BACE1), resulting in abnormal upregulation of these genes, leading to accumulation of Aβ (Kennedy et al., 2004). Moreover, hypermethylation is also observed at a specific position in the promoter of specific genes, such as methylenetetrahydrofolate and apolipoprotein E reductase, in the brain of AD patients. As a result, it can be said that the change of methylation level is one of the causes of Alzheimer's disease related to genes (Wang et al., 2008).

Previous studies also demonstrate abnormal DNA methylation in various psychiatric diseases (Grayson and Guidotti, 2013). Postmortem methylome profiling of brains from patients with schizophrenia has demonstrated intense alterations in the DNA methylation profile, including genes that are related to pathogenesis (Mill et al., 2008). Predominantly, hypermethylation of glutamic acid decarboxylase 67 (*GAD67*) and *RELN* promoter regions was linked to decreased expression of these genes in schizophrenia patients (Guidotti et al., 2000). Table 1.

**Table 1:** The relationship between neurological diseases with genes involved and their methylation.

Neurologic disorders	Methylation status	Specific genetic loci	Probable effect	symptoms
**Rett syndrome**	Unknown	MECP2 gene	Loss of the activity of the MECP2 gene Reduced BDNF (Na and Monteggia, 2011)	Seizures, cerebral palsy, Repetitive, stereotyped hand movements
**Alzheimer’s disease**	Hypomethylation Hypermethylation	PS1, BACE1, APP Neprilysin (NEP)	Upregulation of PS1, BACE1, APP Aβ accumulation (Qazi et al., 2018)	Progressive dementia
**Stroke**	Hypermethylation	Global methylation (Krupinski et al., 2018)		Sudden onset focal dysfunction
**Multiple sclerosis**	Hypomethylation	PAD2	PAD2 Upregulation (Calabrese et al., 2012)	several neurologic symptoms: muscular weakness, visual symptoms, tremors, intestinal and urinary disorders, cognitive abnormalities
**Epilepsy**	Hypermethylation	reelin	Decreased reelin expression (Henshall and Kobow, 2015)	seizure
**Parkinson’s disease**	Hypomethylation	*SNCA* intron1	Increased expression of *SNCA (*Jowaed et al., 2010*)*	Rigidity, tremors, shaking, difficulty in walking
**Immunodeficiency, Centromeric Instability, and Facial Anomalies Syndrome Type1 (ICF1) syndrome**	Hypomorphic mutation of DNMT3B	DNMT3B	Reduced the activity of DNMT3B (De Greef et al., 2011)	Mental retardation syndrome
**ICF2**	Hypomethylation	ZBTB24	hypomethylation of minor satellite DNA and Centromeric instability (Hardikar et al., 2020)	Mental retardation syndrome
**ADCA-DN**	Hypomethylation	DNMT1	reduced DNMT1 activity (Kernohan et al., 2016)	Autosomal dominant abnormality with Cerebellar ataxia
**Amyotrophic lateral sclerosis (ALS)**	Hypomethylation	VEGF, SOD1	No transcriptional silencing (Lu et al., 2013b)	Weakness and atrophy of the muscles
**schizophrenia**	Hypermethylation	GAD67 RELN	Decreased expression of GAD67and RELN (Grayson and Guidotti, 2013)	Hallucinations, Disorganized thinking
**Fragile X syndrome**	Hypermethylation	FMR1	FMR1 inactivation (Kumari et al., 2020)	Intellectual disability
**HSAN1**	Hypomethylation	DNMT1	Reduced DNMT1 activity (Sun et al., 2014)	Multiple neuropathies

MECP2: methyl CpG binding protein 2, BDNF: brain-derived neurotrophic factor, PS1: presenilin 1, BACE1: beta-secretase 1, APP: amyloid precursor protein, NEP: neprilysin or neutral endopeptidase, PAD2: peptidyl arginine deiminase, SNCA: Alpha-synuclein, DNMT: DNA methyltransferase, ZBTB24: zinc finger and BTB domain-containing protein 24, VEGF: vascular endothelial growth factor, SOD1: superoxide dismutase type 1, GAD67: glutamate decarboxylase-67, FMR: fragile X mental retardation, Immunodeficiency, Centromeric Instability, and Facial Anomalies Syndrome Type1; ICF1, Immunodeficiency, Centromeric Instability, and Facial Anomalies Syndrome Type 2; ICF2, Amyotrophic lateral sclerosis; ALS. Amyotrophic lateral sclerosis; ALS.

### 2.2 DNA methylation and depression

Depression is one of the most common psychiatric disorders in the world (Reszka et al., 2021). Depressive symptoms in adolescence have long-term consequences for brain development and can cause severe social and educational problems. Furthermore, depression is linked to cerebrovascular diseases such as stroke (Guo et al., 2021, Xiang et al., 2021). Major depressive disorder (MDD) is a complex, debilitating psychiatric condition with a high prevalence of 3.63%. Symptoms include anxiety, sadness, hopelessness, and emptiness, feelings of guilt or loss and worthlessness, irritability or frustration, loss of interest or pleasure in most routine activities, sleep disturbances, reduced or increased appetite, low energy, difficulty thinking and concentrating, impaired cognition, physical pity, and physical pity. Genetic and environmental risk factors influence depression (Li et al., 2021, Sales et al., 2021, Borçoi et al., 2021). Epidemiologic studies show that genetic factors increase the risk of depression. On the other hand, studies have shown a strong relationship between specific genes and environmental factors in the development of depressive disorder (Sun et al., 2021). Evidence suggests that the onset of depression is increased by around 60% by exposure to stressful events (Sales et al., 2021). Exposure to stress can modify DNA methylation patterns and affects brain plasticity and emotion (Reszka et al., 2021). One of the main neurobiological mechanisms of depression is dysregulated and dysfunctional stress response system (such as hypothalamic-pituitary-adrenal (HPA) axis activity and glucocorticoid receptor (GR) sensitivity) to show the adaptive change (Guo et al., 2021). Moreover, prenatal depressive symptoms might influence fetal epigenetic programming (Kallak et al., 2021). Prenatal depression is associated with differential methylation in GNAS, CTNNA2, OSBPL10, and 5-HTTLPR (Sales et al., 2021, Drzymalla et al., 2021). Mothers with persistent perinatal depression have hypermethylation of OXTR in the saliva. However, this is the only marker associated with perinatal depression in mothers, but no causal effect has been proven (Sales et al., 2021). Maternal depression leads to increased neonatal DNA methylation in the glucocorticoid receptor gene (NR3C1) and BDNF IV promoter (Braithwaite et al., 2015). Preclinical studies have reported stress-induced hypermethylation and reduced gene expression, indicating that exposure to stress conditions in early life leads to persistent epigenetic changes and influences neural and behavioral patterns in adulthood (Sales et al., 2021, Drzymalla et al., 2021). Studies show that epigenetic processes such as DNA methylation are heritable. This could explain the heritability of depression (Juruena et al., 2021). Exposure to stress results in the modification of DNA methylation in several genes associated with depression, including the glucocorticoid receptor (NR3C1 or GR), mineralocorticoid receptor (NR3C2 or MR), corticotrophin-releasing hormone receptor 1 (CRHR1), serotonin transporter (SLC6A4 or 5-HTT), brain-derived neurotrophic factor (BDNF), and FK506-binding protein 5 (FKBP5) (Borçoi et al., 2021, Drzymalla et al., 2021, Ding and Dai, 2019). Different studies have reported the significant relationship between alteration in DNA methylation of FKBP5 with depressive symptoms (Guo et al., 2021, Han et al., 2017, Höhne et al., 2015). These alterations in gene expression may lead to significant modifications in neural and behavioral functions (Sales et al., 2021). Homer1a expression in the hippocampus and cingulate gyrus of patients with major psychiatric disorders including major depression (Leber et al., 2017). Also, Stratum lacunosum glial cells displayed reduced Homer1a expression in bipolar disorder when compared to major depression (Leber et al., 2017). Deletion of synaptic plasticity protein Homer1a results in depression-like behavior and various antidepressant treatments induce its expression (Sun et al., 2021). Gestational stress increases the expression of DNMTs and DNA methylation of BDNF, thereby inducing depressive-like and anxiety-like phenotypes by downregulation of BDNF expression in the hippocampus of the offspring (Zheng et al., 2016).

Also, maternal neglecting or separation stress resulted in hypermethylation of DNA in the hippocampus and the protein phosphatase one catalytic subunit (PP1C) and adenosine A2a receptor (A2AR) promoter in the nucleus accumbens. Upregulation of A2AR is associated with synaptic dysfunction in depression (Carvalho et al., 2019). On the contrary, some studies indicate an association between DNA hypomethylation and stress. For instance, maternal separation stress increased DNA methylation in NR3C1 and Syn I genes, followed by increased NR3C1 mRNA in the hypothalamus, Syn I mRNA, and protein levels in the amygdala, and decreased in the nucleus accumbens (Holmes et al., 2019). Thus, it can be concluded that the alteration followed by DNA methylation depends on various factors, including the type of stressor, age, sex, brain structure, gene, and region (Sales et al., 2021, Drzymalla et al., 2021). Epigenetic modifications of 5-mC and 5-hmC are abundantly found in the brain and are directly associated with depression (Reszka et al., 2021). Transposable elements of Alu and LINE-1, and 5-mC and 5-hmC, have been considered potential biomarkers in mental disorders such as MDD. Interestingly, 5-mC is associated with the downregulation of genes, while 5-hmC is correlated with demethylation and increased transcription (Misiak et al., 2019).

Studies showed a low level of 5-hmC in patients with MDD and a high level of 5-mC in BD type I patients. While in another study on patients with BD and MDD, there was a reduced level of 5-mC and a significant reduction of 5-mC and 5-hmC in major depression (Reszka et al., 2021). In a study by Liu et al., hypomethylation of LINE-1 was observed in the blood of MDD patients (Liu et al., 2016). The mechanism of action of some drugs used as mood stabilizers and antidepressants is based on modifying DNA methylation at specific CpG sites (Goud Alladi et al., 2018).

A review study showed a significant association between DNA methylation and depression risk. Hypermethylation of BDNF, CRMP2, NR3C1, and SLC6A4 is associated with Depression and MDD (Xiang et al., 2021, Li et al., 2021, Borçoi et al., 2021, Li et al., 2019, Schiele et al., 2021, Sanwald et al., 2021, de Assis Pinheiro et al., 2021). Several studies have investigated DNA methylation of some critical genes modulating depressive symptoms, including PTPRN2 (correlated with mood state disturbances), HES5 (associated with MDD and suicide), GATA2 (related to depressive behavior in rats), DGKA (differed significantly between MDDs and controls), NIPA2 (increased risk of MDD), PRDM7 (important in aging and Alzheimer), KCNIP1 (regulate neuronal membrane excitability), GRIK2 (related to mood disorders and depressive symptoms) (Wang et al., 2021). Moreover, HELZ2 and ZNF624 gene expressions differed differentially between MDDs and health controls (Wang et al., 2021). Most patients resist treatment with conventional anti-depressant drugs; according to the results, epigenetic markers can be used in drug responses for psychiatric disorders (Zhou et al., 2021). Characterizing specific DNA methylation patterns identifies novel biomarkers for subtyping psychiatric disorders and the decision of optimal drug choice (Yamagata et al., 2021). For instance, DNA methylation of FKBP5 is a potential marker for the treatment response to mindfulness-based stress reduction in post-traumatic stress disorder (Bishop et al., 2018). Some drugs, such as Clozapine and Sulpiride, activate DNA demethylation in brain tissue. Zhou et al.,2021 found that antidepressant drugs increased DNA methylation in BDNF promoters in patients with MDD and BD (Zhou et al., 2021).

DNA methylation of multiple immune-related loci in patients with depression shows the association between inflammation and depression (Crawford et al., 2018). The study by Sun and colleagues conducted the correlation between promoter methylation of Homer1a and depression-like behaviors. Some antidepressant drugs act through the induction of Homer1a. Moreover, DNA methylation of CpG sites around the binding sites for CRE in Homer1 promoter results in major depressive disorder (Sun et al., 2021).

### 2.3 DNA methylation and ADHD

ADHD is a heterogeneous disorder with a complex and multifactorial background. Numerous genetic and environmental factors and their interactions play a critical role in the pathophysiology of this disease (Barkley, 1998). Recently, genetic risk factors for ADHD have been identified, which include genes involved in neurotransmitter transport, neurodevelopment, growth processes, cell adhesion, and ion transport (Demontis et al., 2019, Rovira et al., 2020). In addition to genetic risk factors, the onset and persistence of ADHD are also associated with environmental factors **(**Jin and Liu, 2018**),** such as low birth weight (Faraone et al., 2005, Karimi-Nazarabad et al., 2015), maternal stress during pregnancy (Humphreys et al., 2019, Palladino et al., 2019), and toxin exposure (Williams and Ross, 2007, Murgatroyd et al., 2009).

Notably, the environment can interact with the genome through epigenetic changes, such as DNA methylation (Murgatroyd et al., 2009, Plazas-Mayorca and Vrana, 2011), which is highly sensitive in early life (Bauer et al., 2016). The role of altered DNA methylation in ADHD has been evaluated primarily through candidate gene studies (Hamza et al., 2019). In addition, the first extensive epigenome communication studies (EWAS) to diagnose ADHD and population symptoms have been performed primarily on relatively small groups of children (Mooney et al., 2020) and adolescents (Meijer et al., 2020).

Epigenetic studies focusing on adult ADHD are rare. Quantitative studies have targeted candidate genes for ADHD, such as norepinephrine transporters (Sigurdardottir et al., 2021), dopamine transporters (Keitel et al., 2018), and serotonin receptors (Perroud et al., 2016). A single EWAS has been performed for ADHD symptoms in the general adult population (Toikumo et al., 2019) and ADHD status (Rovira et al., 2020). Chang et al.’s study of twins showed that the genetic contribution to ADHD varies from childhood to adulthood (Chang et al., 2013), and Meijer et al. Showed that epigenetic differences could distinguish between persistent ADHD and transient ADHD (Meijer et al., 2020). Meijer and colleagues performed targeted bisulfite sequencing for 37 candidate genes to investigate differential DNA methylation between adults with ADHD and healthy individuals. They found that, unlike EWAS, this approach provides information on the methylation level of all CpG sites in target areas (Weiß et al., 2021). More studies are needed to understand better whether people are prone to hyperactivity without a genetic background and simply by the DNA methylation status of neurons.

The results of the studies showed that the further analysis of DNA methylation in ADHD can help identify the biomarkers of the disease and potentially the mechanisms of the disease, with the results of some studies pointing to the relationship between the methylation level of promoter of *GART* and *SON, SLC7A8, MARK2, ERC2 and CREB5* genes (Mooney et al., 2020, Neumann et al., 2020) and DRD4 and 5-HTT regions (van Mil et al., 2014) and the disease. Eventually multi-positional algorithms will be essential for discovery of clinically valuable biomarkers.

### 2.4 DNA methylation and Alzheimer’s disease

AD is the leading cause of dementia and also one of the most pressing public health issues in our life. By 2050, it is anticipated to have reached a global prevalence of over 91 million AD cases. Although the pathogenesis of AD is yet unknown, the most commonly recognized theory is the amyloid pathway, in which the accumulation of tangles and plaques is claimed to play a crucial role in the disease’s course and development (Ozaki and Niida, 2019).

However, other characteristics, including phospholipid metabolism, cholesterol, and abnormal calcium, frequently appear before the accumulation of tangles and plaques appear early in disorder. The analysis of genome set and disorder cascade analysis obtained from the findings of “epigenome-wide association studies (EWAS)” proposed biological functions involving the amyloid-β protein precursor (APP) degradation, tau adhesion molecules, lipid-related mechanisms, and brain immune functions in the pathogenesis of Alzheimer’s disorder (Wei et al., 2020). Moreover, AD is seen as a multifaceted illness that results from the combination of genetic and environmental variables, which are influenced by epigenetic processes (Altuna et al., 2019b).

There is increasing evidence that epigenetic variation plays a significant role in the development of Alzheimer’s disease, although gene mutations account for just 5% of all cases. Furthermore, recent methodological developments can employ EWAS in complex disorders phenotypes, such as AD. Epigenetics reversibly regulates gene expression and may be inherited through cell division (Prasad and Jho, 2019). DNA methylation is a vital epigenetic pattern that manages changes in specific genes and helps regulate gene expression in vertebrates, which is the best-studied example of epigenetics modifications in AD (Wei et al., 2020).

The link between AD and DNA methylation has been studied extensively. In the peripheral blood and brain, distinct methylations of genes were discovered in control groups and AD patients. The APP gene was the only one that was consistently hypermethylated in both the blood and the brain, suggesting that it might be the most effective diagnostic biomarker of blood for AD. Furthermore, there was an increase in the APP gene expression in AD patients (Wei et al., 2020, Iwata et al., 2014). In addition, Coppieters et al (Coppieters et al., 2014). found a positive correlation between global levels of 5mC and amyloid-beta in the brain of patients with AD.

An in vitro investigation has shown that APP hypermethylation is related to higher expression in AD brains. Although other studies reported no change in relative hypomethylation in various areas of the APP gene in individuals with AD, the methylation-detecting techniques utilized in this research were not sufficiently sensitive, affecting the credibility of the findings. This finding will need further investigation to be confirmed (Wei et al., 2020).

Altuna et al (Altuna et al., 2019b). proposed that altered methylation of DNA in the AD hippocampus happens at particular regulating areas that can be critical for neuronal differentiation, supporting the idea that adult hippocampus neurogenesis may have a role in the development of AD.

Several genes have been discovered to be differentially methylated in AD brain autopsy samples using “Illumina Infinium Human Methylation450K arrays”, including those genes previously identified as carrying genetic variations for AD, such as BIN1 (amphiphysin II) or ANK1 (ankyrin-1) (Altuna et al., 2019b, Lunnon et al., 2014).

Interestingly, some of these DNA methylation patterns are available in the early AD stages, indicating that such modifications may play a role in the disease’s development. Overall, these studies add to our knowledge of the pathophysiology of AD (Altuna et al., 2019b).

### 2.5 DNA methylation and Rett syndrome

Rett syndrome (RTT) is a common mental disability that occurs once per 10,000–22,000 girls. It is marked by a stage of average growth and development until approximately one year, followed by a fast regression that includes stereotypic hand wringing, irregular breathing, ataxia, autism, slowed head growth or microcephaly, lack of acquired motor and verbal abilities, seizures. Despite these symptoms, patients live until maturity (Kriaucionis and Bird, 2003).

According to a recent study, mutations of MeCP2 cause RTT syndrome (MIM 312750), a juvenile neurological illness that is amongst the most prevalent due to mental impairment in women. Extensive RTT patient screening indicated that 80% of patients with RTT syndrome are related to detectable mutations in MeCP2 gene, including insertions, deletions, nonsense, and missense (Goffin et al., 2011, Kriaucionis and Bird, 2003). Moreover, MeCP2 is overexpressed in the postnatal brain, suggesting that methylation-dependent gene regulation can play an essential role in the development of the mammalian central nervous system (Chen et al., 2001). Furthermore, several genes become silenced when the promoters of these genes are methylated. Hence, using transient transfection research, scientists assumed that *MeCP2* gene was a transcriptional suppressor and could suppress the transcription in both cells and in-vitro (Esteller, 2008, Petralla et al., 2021). To investigate MeCP2’s suppression properties, scientists monitored reporter gene expression with a fusion of the GAL4 DNA- binding domain to the various parts of *MeCP2* gene (Petralla et al., 2021). A domain with 100 amino acids was observed in the middle of the protein, which is in charge of transcriptional suppression (TRD).

Additionally, it revealed that the binding of *MeCP2* was capable of suppressing the transcription (from up to 2000 bp of the transcription start site (TSS)) (Kriaucionis and Bird, 2003). The DNA electrophoretic mobility shift assay (EMSA) was utilized by W. Gabel et al (Gabel et al., 2015). to evaluate the *MeCP2* binding to different forms of methylated DNA. Consistent with similar studies, MeCP2 showed high affinity to mCG DNA and not hmCG, which proves that *MeCP2* might not preferentially bind to hmCG in neurons. In contrast, *MeCP2* binds to mCA, hmCA, and mCG with high affinity compared to binding to mCC and mCT with low affinity (the same affinity to unmethylated DNA), respectively. This tight binding between *MeCP2* to mCG, mCA, and hmCA indicates the potential role of *MeCP2* in regulating long gene expression in the brain through binding to the referred sites. Also, a thin-layer chromatography, and Tet-assisted bisulfite sequencing (TAB-seq) analysis, showed that the methylation form of hmCA is rare in the brain (Gabel et al., 2015).

The frequency of hCG and mCH in the neuronal genome and the level of *MeCP2* protein were significantly increased over the postnatal period. As such, this suggests that *MeCP2* could play a role in the maturation of neurons by binding to hmCG and/or mCH methylated DNA (Szulwach et al., 2011, Kriaucionis and Heintz, 2009).

### 2.6 The role of DNA methylation in Therapeutic approaches to neurological diseases

Epigenetically targeted drugs, in general, and DNA methylation-targeted drugs, in particular, may have distinct pharmacological and toxicological properties. DNA methylation is the best-studied epigenetic mechanism in eukaryotic cells. Mutations in genes can cause epigenetic dysfunction leading to certain neurodevelopmental disorders (Stirzaker and Armstrong, 2021). Some altered epigenetic patterns are directly associated with the presence of a mutation in an epigenetic gene involved in a neurodevelopmental disorder. It has been reported that DNA methyltransferase activity is high in neurons, and its activity may contribute to induced ischemic brain damage in mice (Dolen et al., 2019). DNA-demethylating drugs are currently considered as a treatment option (Nuzziello and Liguori, 2021). These drugs may be suitable for various neurodegenerative and neurodevelopmental diseases, such as fragile X syndrome. Histone deacetylase (HDAC) inhibitors are the recent focus for researchers (Kumari et al., 2020). Epigenetic mechanisms are a central process in determining cell fate (Sivalingam and Samikkannu, 2020). However, there are no new epigenetic regulators of development nor known mechanisms to be used for development. This is a rich area for additional research, especially regarding noncoding RNAs and their role in CNS development (Sivalingam and Samikkannu, 2020).

All current approaches to modifying DNA methylation levels target the endogenous enzymatic machinery responsible for adding and removing mCs from DNA in some way. The use of constitutive and conditional gene knockout mouse models and viral-mediated RNA knockdown or overexpression techniques has revealed much about the importance of active DNA methylation during neurodevelopment and in the functioning adult CNS (Kaur et al., 2022). The contribution of the epigenome in protection against neurodegenerative diseases such as AD or PD have been demonstrated (Suchy et al., 2010). For example, supplementation of S-Adenosyl methionine (SAM) in a transgenic mouse model (SOD1-G93A) of amyotrophic lateral sclerosis (ALS) delayed the onset of motor neuron pathology. HDACi also facilitated disease progression in ALS animal models (Suchy et al., 2010). Sodium phenylbutyrate significantly extended survival in G93A transgenic ALS mice (Ryu et al., 2005).

Research into Huntington’s disease (HD), a neurodegenerative disorder caused by a trinucleotide repeat expansion in the gene (HTT) encoding the huntingtin protein, found that mutant huntingtin interacts directly with HAT proteins, resulting in altered histone acetylation (Jiang et al., 2006). Numerous studies have shown that treatment with HDACi halts progressive neuronal degeneration in both fly and mouse HD models. Several selective HDACi and other compounds are investigated (Chopra et al., 2012, Duan, 2013).

Researchers now know that, while DNMT3a/b are frequently responsible for de novo methylation and DNMT1 for its maintenance, these roles are not mutually exclusive, and knocking out both DNMT1 and DNMT3a in adult forebrain neurons is required to elicit dysfunction in long-term plasticity and deficits in learning and memory (Mattei et al., 2022). A Tet1 knockout mouse model and RNA knockdown experiments recently demonstrated that Tet1-mediated mC oxidation is required for memory and the regulation of activity-related genes in the dorsal hippocampus, including Fos and Arc (Zhan et al., 2022). Previous studies have shown that targeting the epigenome, especially with small drug molecules, can cross the blood-brain barrier and delays the onset and progression of symptoms in animal models of neurodegenerative disease (Fischer, 2014). As suggested in many reports, the multicentric and multicellular effects exerted by most drugs, the possibility of unexpected side effects, and the anatomic and metabolic differences between humans and rodents are reasons for concern (Fischer, 2014). This suggests that further studies are needed to clarify the most appropriate therapeutic approaches, including the use of selective inhibitors, timing, dosing regimen, a better understanding of the interplay between histone tail modifications and other mechanisms regulating gene expression, and evaluation of potential side effects (Fischer, 2014). In addition, the route of drug administration varied across studies, are not expressed in the same way in brain regions affected by AD, PD, or other neurodegenerative diseases. Although HDAC2 and HDAC6 may represent promising drug targets in AD, it remains unknown which drug or dosing regimen is most effective, and similar conclusions can be drawn for other neurodegenerative diseases (Harrison and Dexter, 2013).

**Fig. 2:**
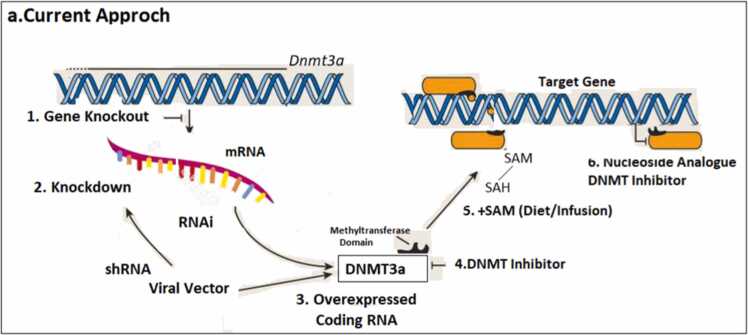
Current Approaches to manipulating DNA methylation.

Indeed, while recent tools such as fluorescence-activated cell sorting and next-generation sequencing have greatly improved the ability to measure epigenetic changes with cellular and genetic precision (see sidebar, Measuring the Epigenome), our approaches (Fig. 2) to manipulating DNA methylation are far less sophisticated. In contrast, as demonstrated for various DNMT proteins and Tet1, genetic processes such as traditional gene knockout animal lines, small hairpin RNA knockdown, and virally mediated gene overexpression are capable of exhibiting complete isoform selectivity (Sagarkar et al., 2022).

These approaches still have limitations because they do not provide precise temporal control over methylation status. Furthermore, because they presumably affect methylation status on a genome-wide scale, these approaches still lack target specificity. Another limitation to our knowledge is that the drugs have been tested in animal models carrying mutations in human genes such as SOD1, which account for only about 1–2% of human cases. In addition, histone code writers or deletion proteins could be potential drug development targets, but studies are limited. Natural compounds found in the diet, including folate, vitamins, polyphenols, and flavonoids, alter the availability of methyl groups and affect the activity of DNMTs, thus representing potential “epigenetic” preventive factors for neurodegeneration.

## 3 Conclusion

In this brief overview, we reviewed the emergence of a new understanding of epigenetic molecular mechanisms and their implications for CNS function and dysfunction. Studies have shown how various environmental factors, such as stress, smoking, radiation, diets, and medications throughout life can affect epigenetics and thereby act as strong determinants of human health in the coming decades. Research in the previous two decades has discovered an emerging outline of the relationship between numerous epigenetic pathways and neurological disorders. However, changes in chromatin structure are probable to happen in many loci of the genome, and it is imperative to conduct epigenetic studies at the genome level to examine this issue in animal models like the Alzheimer’s model.

Perhaps the results of this study and the studies mentioned can lead to advances in treatment approaches and tools needed by person-centered medicine in neurological disorders. Therapeutic approaches aimed at creating of global epigenomic maps in neurological disorders in histone modification patterns, DNA methylation, and RNA expression in primary tissues and cell types of all major lineages in the human cell body would be valuable. Finally, it appears that the various epigenetic changes do not occur separately and that DNA methylation and histone modification changes occur side by side and affect each other. A complete understanding of these epigenetic changes and their interactions will lead to better treatment strategies for neurological disorders such as hyperactivity and mental health problems in patients with ADHD, Alzheimer’s disease, stress, and depression. By studying the mechanisms and targets of epigenetics, especially DNA methylation, drug modulation and regulation of epigenetic processes, there will be many promises for the treatment of neurological diseases.
